# Upscaling Arbuscular Mycorrhizal Symbiosis and Related Agroecosystems Services in Smallholder Farming Systems

**DOI:** 10.1155/2016/4376240

**Published:** 2016-01-28

**Authors:** Marjorie Bonareri Oruru, Ezekiel Mugendi Njeru

**Affiliations:** Department of Microbiology, Kenyatta University, P.O. Box 43844-00100, Nairobi 00100, Kenya

## Abstract

Smallholder farming systems form unique ecosystems that can protect beneficial soil biota and form an important source of useful genetic resources. They are characterized by high level of agricultural diversity mainly focused on meeting farmers' needs. Unfortunately, these systems often experience poor crop production mainly associated with poor planning and resource scarcity. Soil fertility is among the primary challenges faced by smallholder farmers, which necessitate the need to come up with affordable and innovative ways of replenishing soils. One such way is the use of microbial symbionts such as arbuscular mycorrhizal fungi (AMF), a beneficial group of soil microbiota that form symbiotic associations with majority of cultivated crops and play a vital role in biological soil fertility, plant nutrition, and protection. AMF can be incorporated in smallholder farming systems to help better exploit chemical fertilizers inputs which are often unaffordable to many smallholder farmers. The present review highlights smallholder farming practices that could be innovatively redesigned to increase AMF symbiosis and related agroecosystem services. Indeed, the future of global food security depends on the success of smallholder farming systems, whose crop productivity depends on the services provided by well-functioning ecosystems, including soil fertility.

## 1. Introduction

Agriculture and food supply in several developing nations have been reshaped over the last few years partly due to the growing push towards industrialization and globalization. This push emphasizes on export crops and biofuel crops, which have been broadly embraced worldwide. Although such practices have led to a short-term productivity increase, the long-term ecological impacts and risks associated with them have been overlooked [1]. Regardless of these emerging trends, smallholder farming systems (SHS) that comprise an array of ecologically based agricultural forms offer a high potential for promoting biodiversity and sustaining yield with minimal chemical-based agricultural inputs. They conserve ecological integrity while providing sufficient agricultural output for domestic consumption in numerous countries.

Smallholder farmers significantly contribute to global food production. These farmers supply up to 50% of the world's cereal, 60% of global meat, and 75% of global dairy production [2]. Both rural and urban food consumers in developing nations heavily rely on the efficiency of local smallholder farmers to meet their subsistence needs. Smallholders are, therefore, gross domestic food and nutrient providers and play a crucial role in the world's effort to improve nutritional and food security. Unfortunately, smallholder farmers have not been the focus of agricultural development until recently [3]. Failure to focus on such farmers has resulted in a number of challenges that have been a major hindrance to food production.

Smallholder farming systems generally experience poor crop production and perennial food insecurity. This is especially the case in the semiarid tropics where many smallholder farmers reside [4]. In addition to inadequate rainfall, decline in soil fertility resulting from inappropriate soil management practices is a major crop production constraint. This is worsened by additional factors like inappropriate land use policies, land availability constraints, and failure to invest in agricultural research. Other constraints to productivity in such systems include failure to fully utilize organic resources, nonsupportive institutions, and harsh climatic conditions, especially in sub-Saharan Africa (SSA) agroecosystems [5].

Most of the smallholders farming systems in semiarid regions are faced by a challenge of soil fertility management. They have minimal concentrations of nutrients such as phosphorous and nitrogen [6]. Small scale farmers have for several decades removed large quantities of nutrients from their soils without using sufficient quantities of fertilizers to replenish the soil. This has led to an average annual depletion rate of 22 kg of nitrogen, 2.5 kg of phosphorous, and 15 kg of potassium per hectare of cultivated land in 37 African countries over the last 30 years [7]. The traditional way of overcoming nutrient depletion is the use of mineral fertilizers, which are unfortunately too costly for the majority of smallholder farmers.

There has been a surging interest in ecofriendly and sustainable agricultural practices [8] such as the use of microbial biopesticides and biofertilizers. In addition to enhancing plant growth and productivity, such products provide other beneficial ecosystem services that sustain the environment. Microbial based formulations could thus be adopted by smallholder farmers to serve as a cheap and efficient way of enhancing soil fertility. Although the price of initial inoculum could be high, low cost multiplication technologies, especially on-farm multiplication, would significantly lower the cost. There are diverse types of efficient microbes in the rhizospheric soils, such as arbuscular mycorrhizal fungi (AMF) that have beneficial effects on plant productivity.

AMF are beneficial soil microbes that belong to phylum Glomeromycota [9] and that form symbiotic associations with majority of wild and cultivated plant species. Fossil and molecular evidence has shown that this group of fungi has been in existence for more than 400 million years [10]. This is a clear indication that AMF have been through a long coevolution period and may indicate a great selective symbiotic advantage for both the plant and the fungus [11]. AMF play a vital role in plant nutrition and soil fertility [12] and could thus be of great benefit which include increased nutritional uptake, productivity, and improved yield quality to many crops grown by smallholders across the world (Table 1).

While the fungus gains by obtaining photosynthetic carbohydrates from the host plant, the host plant gets nutritional and protective benefits. In this review paper, we analyze some of the characteristics of SHS and how they influence AMF symbiosis. We also discuss how AMF are an essential resource that could be efficiently utilized by smallholder farmers to enhance long-term soil fertility and crop production.

## 2. Elements of Smallholder Farming Systems

**Figure 1:**
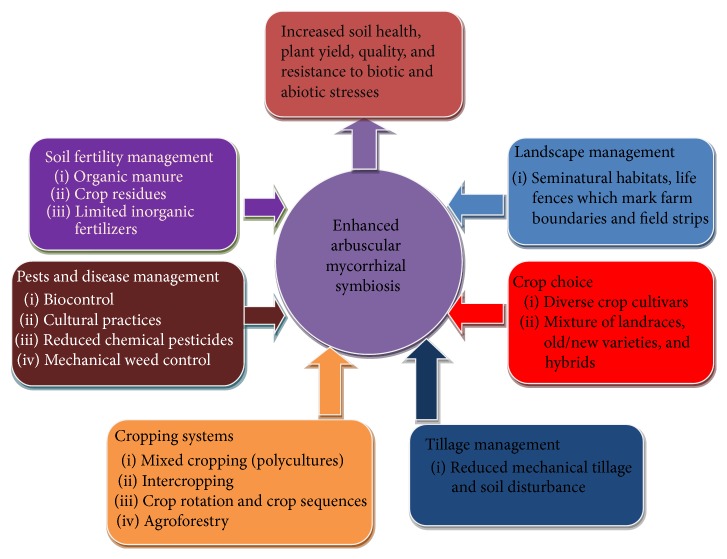
Key elements of smallholder farming systems (SHS) that enhance arbuscular mycorrhizal symbiosis and related agroecosystem services. SHS are characterized by high agricultural biodiversity because of practices such as agroforestry, intercropping, and diversified crop cultivars. Since smallholder farmers rarely use chemical farm inputs such as fertilizers and pesticides, most opt for organic manure, a cheap alternative. Such practices enhance mycorrhizal symbiosis which in turn increases soil health, plant yield, quality, and resistance to biotic and abiotic stresses.

Smallholder farming systems are prevalent in developing countries and are characterized by small-sized farms with complex farming styles (Figure 1). Farmers have adapted these farming styles depending on the local conditions to help them sustainably manage harsh environments and meet their dietary needs without intensively relying on mechanization and chemical farm inputs. Over the years, peasants across the globe have displayed a high degree of innovation, which has led to success of indigenous agriculture. There are more than three million hectares under peasant agriculture that incorporate practices such as agroforestry, mixed farming polycultures, and many more [3].

A key feature of smallholder farming systems is high biodiversity levels in form of agroforestry and/or polycultures (Figure 1). Several crop species and varieties are planted within the same piece of land and this leads to long-term stabilization of yield, maximizes returns even in low technology, and promotes diet diversity. Such biodiversity also increases the number of insect predators, pollinators, nutrient-enriching plants, nitrogen-fixing bacteria, and other beneficial microbes. Smallholder agroecosystems contain populations of different landraces which are genetically diverse, hence providing security to farmers against droughts, pests, diseases, and other biotic and abiotic stresses. The stability of cropping systems is heightened by genetic diversity and this allows farmers to exploit various microclimates and derive multiple nutritional uses [43]. Therefore, diversity in smallholder farming systems plays a fundamental role in regulating the functioning of an ecosystem.

Another key feature of SHS is their resilience amidst continuous economic and environmental changes, while substantially contributing to local, regional, and national food security [44]. For this reason, agroecologists have noted that smallholder agroecosystems have the capacity to provide solutions to several uncertainties encountered by humanity in the face of climate change and financial and energy crisis. Smallholder agricultural systems exist in several countries including Africa, Asia, and Latin America where they form an essential ingenious agricultural heritage reflecting the value of agricultural systems diversity adapted to variable environmental conditions. Unfortunately, the future of these farming systems is not guaranteed, especially with modern agriculture emphasizing on intensive tillage and the use of chemical farm inputs [2].

SHS have unique cropping systems with practices such as agroforestry, crop rotation, and intercropping being dominant (Figure 1). To meet their subsistence needs, these farmers opt to grow a diverse number of crops in their small-sized farms. These practices have numerous benefits; for example, there are studies that have been done to evaluate the advantages attributed to intercropping [45]. Intercropping is beneficial to plants in the context of preventing soil erosion, improving soil texture, promoting better water penetration, resource use efficiency, and supplying organic matter in addition to promoting the colonization of symbiotic microbes such as indigenous AMF in soil (Figure 1). A recent meta-analysis of 290 published glasshouse and field trials to investigate the effects of various agricultural practices on mycorrhizal colonization led to the finding that inoculation increased mycorrhizal colonization by up to 29% [46]. Therefore, the use of AMF inocula could be of importance to smallholder farmers in SSA who often farm on low fertility soils and have minimal or no access to mineral fertilizers.

## 3. Arbuscular Mycorrhizal Fungi as a Resource in Smallholder Farming Systems

Smallholder farming systems are identified by low input cropping systems where the natural activities of microbes contribute to biocontrol of plant pathogens and improved nutrient supply, thus maintaining crop health and production. Symbiotic mycorrhizal fungi such as arbuscular mycorrhizal fungi form a primary component of microbial populations that form symbiotic association with higher plants, thus influencing the plants' growth and productivity [47]. AMF are multifunctional in nature and may play a crucial role in dissolution, weathering and cycling of mineral nutrients [48], carbon cycling, nutrient mobilization from organic substrates, and mediation of plant responses to different environmental stresses like heavy metal toxicity, soil salinity, heat stress, drought, plant pathogens, and soil acidification. Intensive agriculture that involves tillage and high farm inputs has led to marginalization of the natural roles of these microbes [49]. Hence, development of sustainable soil fertility management and crop production would necessitate a better understanding of the microbial interactions.

**Figure 2:**
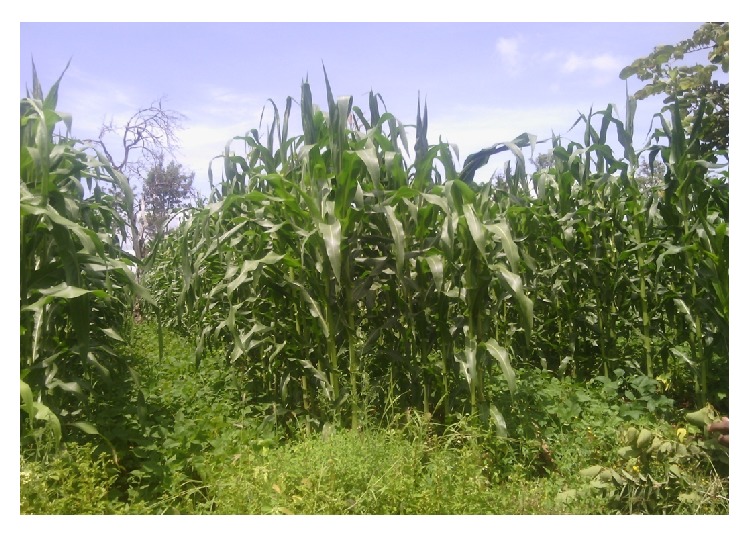
A smallholder farming system in Mbeere North, Kenya, showing a maize-common bean intercrop, under reduced tillage cultivation, which also incorporates agroforestry. Legume-rhizobia symbiosis fixes nitrogen which enhances growth of both the legume and the cereal crop. Arbuscular mycorrhizal symbiosis is enhanced which promotes nutritional uptake by both crops and also increases N_2_ fixation by availing P. Agroforestry trees most of which are legumes that provide host to the beneficial soil microorganisms especially after farmers harvest the other crops.

In mycorrhizal symbiosis, the role of the plant's root hair is complemented by the fungus, which acts as an extension of the root system [50]. Mycorrhizal colonization increases the absorption surface area, exposes greater soil areas, and increases the life-span of absorbing roots. In this way, soluble nutrients are better utilized and retained because of reduced reaction with soil colloids or leaching losses [51]. Nodulation and atmospheric nitrogen fixation potential in legumes are also increased by AMF [52]. This is because AMF improve phosphorous uptake by the plant, which in turn would avail more energy for nitrogen fixation by rhizobia (Figure 2). Thus, dual inoculation of plants with rhizobia and AMF would show synergistic effects on nodulation and nitrogen fixation [53]. Mycorrhizal colonized roots are also highly likely to be colonized by other microbes, and their susceptibility to soil-borne pathogens such as phytopathogenic fungi or nematodes is lowered [51].

AMF alter the soil-plant-water relations and, in this way, plants become better adapted to adverse conditions like salinity, drought, or heat stress. Mycorrhizal fungi have been shown to alleviate the toxicity of heavy metals thus allowing plant growth [54]. The real value of mycorrhizal fungi is that they form a link between plants and heterogeneously distributed nutrients needed for their growth. Hence, they enable the flow of energy-rich compounds necessary for nutrient mobilization while at the same time providing a way through which the mobilized products are transported back to their hosts. It is therefore essential to understand the ecology and functioning of arbuscular mycorrhizal symbiosis with the aim of improving their functionality in SHS.

## 4. Agroecological Practices That Support Mycorrhizal Symbiosis in Smallholder Farming Systems

Agroecological practices range from advanced technology-based to ecology-based practices. While precision farming or utilization of genetically modified crops could help meet the future nutritional demands, other practices like natural biological pest control and reduced tillage could increase the activity of soil microbes and improve soil fertility [55]. Some of the well-known agricultural practices that have been widely adopted in SHS include biological pest control, crop rotations, organic crop fertilization, crop rotations, intercropping, agroforestry, livestock integration, and other biodiversity conservation practices [7].

The practices in question are related either to the management of landscape elements or to crop management. Crop management practices include those that address crop spatial distribution, crop choice, and crop temporal successions. They also include fertilization practices, irrigation practices, tillage practices, and weed, pest, and disease management practices [56]. The question of diversification is inevitable in developing agroecological practices. In the last decade, emphasis has been laid on reintegrating species diversity into cropping systems for various reasons. For instance, it leads to decreased pest outbreaks or biodiversity conservation [57]. Diversification entails integrating different crops, cultivars, or intercrops into cropping systems. It is evident that the choice and design of agroecological practices not only promote agricultural sustainability but also enhance microbial activity such as AMF symbiosis as will be highlighted below.

## 5. Soil Fertility Management

Soil fertility is one of the major challenges that smallholder farmers encounter. Most of the soils in such farming systems are low in nutrients such as P and N since farmers are not able to replenish soils using costly chemical fertilizers. Chemical fertilization is an agricultural practice that poses threat to AMF symbiosis. The presence of high levels of chemical fertilizers in soil not only leads to environmental drift and possible pollution of underground water reservoirs but also alters the association between microbial communities and plants. The vital role of AMF in plant nutrition makes them very sensitive to changes in availability of soil nutrients. Basically, a nutrient-rich environment allows the plant to take up sufficient nutrients without relying on AMF symbionts. This results in a gradual reduction of the plants' dependency on their AMF partners and a consequent decline in AMF community richness and diversity [58, 59].

Biofertilizers refer to substances that contain living microbes, which when applied to plant surfaces, seed, or soil colonize the interior of the plant and promote growth by increasing the availability of primary nutrients to the host plant. AMF are among the major group of microbes that are considered as biofertilizers or bioenhancers [8]. They form a mycelia network that increases the magnitude of soil volume which can be explored by a plant. In this way, a mycorrhizal root would be more efficient in phosphate uptake compared to a nonmycorrhizal root [12]. Several studies have demonstrated the beneficial effect of AMF on increasing the tolerance of plants to biotic stress caused by the interaction of soil-borne pathogens with different plant species. AMF have been shown to suppress pathogenic fungi such as* Rhizoctonia*,* Fusarium*,* Thielaviopsis*,* Verticillium*,* Pythium*,* Aphanomyces*, and* Phytophthora* [60].

Smallholder farmers could adopt AMF as biofertilizers to promote soil fertility. The application of such microbes would promote nutrient uptake efficiency and thus minimize the use or need for costly phosphatic fertilizers. Additionally, the use of AMF on SHS would greatly reduce root infection and disease severity caused by pathogens, and this results in increase in both crop yield and quality. Mycorrhiza can thus be termed as a health insurance for plants [61]. Even when there is no immediate positive effect on yield and plant growth, a reduction in disease development would decrease pathogen populations in the soil and this may have a beneficial impact on the following crops. Generally, the high tolerance of mycorrhizal plants against root pathogens provides bioprotection as an ecosystem service for sustainable agriculture.

AMF development is characterized by formation of an extensive mycelial network into the surrounding soil. This network can account for up to 50% of the total fungal mycelium in the soil, thus representing a major part of soil microbial biomass [61]. The external hyphal network enmeshes soil particles, consequently improving their structure. Additionally, the presence of AMF has often been correlated to increased level of glomalin related soil protein (GRSP) which binds soil particles together thus contributing to water retention and soil fertility [20, 62]. The extensive hyphal network coupled with GRSP helps in stabilization of soil aggregates, thus leading to enhanced soil structural stability and quality.

## 6. Crop Succession and Spatial Distribution

A classic way of introducing crop diversity in SHS is through redesigning crop spatial distribution and temporal successions. Crop spatial distribution entails the management of intercropping and agroforestry practices to optimize synergies and positive interactions between crops. Due to scarcity of space and need for diverse nutrition, many smallholder farmers intercrop legumes and cereals while incorporating agroforestry trees (Figure 2). Interestingly, this practice does not only ensure a more balanced diet for the farmer but also promotes soil health. Through tripartite symbiosis, the legume-rhizobia crop fixes nitrogen and provides adequate carbon to AMF which in return provides nutritional benefits especially P, a key nutrient for N_2_ fixation. Nonetheless, different legumes and cereals may differ on how they interact with rhizobia and AMF. In the last two decades, a lot of research has been conducted to enhance rhizobia-legume symbiosis among smallholder farmers [63].

In 1980s, the concept of the Microbiological Resources Center (MIRCEN) was advocated and developed by UNEP with one of the main centers based in Nairobi, Kenya. The main objective of MIRCEN, Nairobi, was to address N_2_ fixation by legume-rhizobia systems including the identification and development of efficient rhizobia cultures compatible to crops in SSA [64]. Since then, rhizobia inoculants commonly known as Biofix have been developed and are currently adopted by farmers [65]. Moreover, in the 1980s, the International Institute for Tropical Agriculture (IITA) developed promiscuous soybeans which would fix nitrogen with diverse rhizobia communities across different agroecological zones [66]. Despite all the aforementioned efforts, poor legume nodulation has been reported in many studies. One common reason could be the limited knowledge and use of AMF symbiosis by many farmers since we know that there is a close and strong rhizobia-AMF-legume interaction. One proposed reason for poor N_2_ fixation in the tropics is the acidic nature of soil and P fixation. Interestingly, AMF would play a key role in availing P while promoting tolerance to low pH. Furthermore, the overall outcome of AMF-rhizobia symbiosis in intercropped systems will be influenced by the legume and cereal crops. Owing to the high diversity of crop cultivars in SHS, there is the need to screen these cultivars to identify those that favor positive interactions with beneficial microbes.

A way of promoting crop diversification is through crop rotations and cropping sequences that incorporate mycotrophic crops. For instance, integrating legume crops into a rotation will not only enhance atmospheric nitrogen fixation but also promote AMF symbiosis and related ecosystem services. Besides, bare-soils or soils planted with nonmycorrhizal crops have lower AMF spores and hyphal density, as well as lower AMF infectivity [67]. Mycorrhizal infectivity and spore densities in the soil may be influenced by the identity of the crop species. For instance, a study conducted by Troeh and Loynachan [68] showed that a maize field contained higher mycorrhizal spore density compared to a soybean field. In Niger, it was demonstrated that sorghum roots had 10–15% higher AMF colonization when rotated with either groundnuts or cowpea than if grown continuously [69]. It is thus clear that crop rotation greatly affects both the composition and diversity of AMF spore communities in the soil, with rotated crops having higher AMF diversity than monocultures [70].

The magnitude of symbiotic benefits conferred to crops may be affected by the changes in infectivity and composition of AMF communities caused by crop rotation. For example, maize rotated with sunflower or soybeans had a higher dry-matter production and *P* uptake compared to maize grown under bare-soil fallow [71]. Different AMF species have been found to have different affinities towards specific host plants. This specificity could explain why different crop-plant species affect AMF composition and diversity. More diverse AMF communities can therefore be established in soils in an intercropping and crop rotation system that considers more plant species [72]. It is thus vital for SHS to have better planning of these sequences to maximize on the benefits conferred by AMF. For example, intercropping of mycotrophic crops with nonmycotrophic crops will ensure that the beneficial effects of AMF are attained by all crops.

## 7. Cover Crop Modification

Cover crops are generally valued as an essential management practice for sustainable agriculture because of the role that they play in soil conservation and quality, weed suppression, and crop performance [73, 74]. Some of the agroecosystem services that smallholder farmers can gain by incorporating cover crops into the soil include control of soil erosion and nutrient leaching, supply of plant nutrients, interruption of pest, disease and weed cycles, and maintenance of soil biodiversity [75]. In addition, cover crops are mainly important in the replacement or supplementation of inorganic N fertilizer, through N fixation by leguminous cover crops or scavenging of residual available N by cereal cover crops or microbial decomposition of cover crop residues [76, 77].

Cover crop management is dependent on their intended use, whether as green manure, living mulch, or dead mulch. In the Mediterranean region, winter cover crops are seeded in late summer or early fall and maintained in the field through winter and spring. Towards the end of spring, the cover crop biomass is either destroyed or incorporated into the soil by cultivation or ploughing (green manuring) or mowed and left on the soil surface as a dead surface mulch [78]. Although ploughing decreases the exposure of biomass to air and atmospheric agents, it usually enhances microbial decomposition of the cover crop biomass compared to surface mulching. Incorporation of cover crops into the soil through deep ploughing may also negatively affect AMF symbiosis [79], although this aspect has not been critically investigated.

Cover crops may indirectly affect crop productivity by influencing rhizospheric soil microbiota, particularly AMF [80, 81]. Since AMF are obligate mutualists, cover crops maintain or increase soil mycorrhizal potential by providing them with nourishment, especially during winter periods [73, 82]. However, some cover crops (e.g.,* Brassica* spp.) are mycorrhizal nonhosts and additionally produce mycotoxic compounds from degradation of glucosinolates upon tissue disruption and thus may negatively affect AMF communities. Until now, there are conflicting reports, either negative [83, 84] or neutral [85, 86] on the effects of* Brassica* crops (either as cover crop or main crop) on soil mycorrhizal potential and root colonization of the subsequent crop. Thus, for cover crops to be incorporated into SHS to ameliorate AMF populations, it would be necessary to have comparative field experiments that encompass AMF host and nonhost cover crops plus fallow.

The intended benefits of cover crops depend on the cover crop species, composition, prevailing environmental conditions, and field management. Cover crops can be grown as monocultures or as species mixtures, where the latter aim to optimize resource use efficiently and the associated agroecosystem services through increased diversity. Although there is considerable documentation on the use of single cover crop species [74, 87], relatively little information is available on cover crop mixtures, especially in SHS. Cover crop diversification may increase the aboveground biomass, the amount of N fixed, weed suppression, and soil biodiversity and promote timely decomposition of the cover crop biomass depending on the crop needs by more equilibrate C : N ratios [88]. Moreover, cover crop mixtures may be more tolerant to adverse environmental conditions than monocultures, hence promoting resilience, which is vital, particularly in the current era of unpredictable weather conditions.

There is a need to use mycorrhizal responsive cover crops in smallholder farming systems to maintain AMF propagules all year round. The effect of AMF is determined by plant genotype. Specific plant species have specific influence on development of AMF, and it seems that plant species incorporated as cover crops may, primarily, determine the composition of AMF populations thriving in the soil [89]. It is thus essential to identify and constantly use mycorrhiza-responsive crops as a buffer to loss of biotrophic mycorrhizas.

## 8. Tillage Management

A shift from conventional to reduced tillage or no tillage helps in minimizing energy consumption, decreasing wind and water erosion, reducing soil compaction, increasing soil biota activity, and increasing organic matter and subsequently carbon sequestration. No tillage involves practices such as direct seeding into dead or living mulch, which does not result in soil disturbance. Reduced tillage corresponds to reduced soil disturbance without soil invasion, which is contrary to ploughing. The soil is worked to a depth of 5–15 cm prior to seeding. The main objective is to minimize soil disturbance, preserve organic matter at the soil surface, and improve soil fertility [55]. Reduced or no tillage practices are currently being adopted globally, including tropical regions [90].

The practice helps in lowering energy inputs hence increasing the efficiency of the cropping system. Other benefits include minimizing soil erosion, stocking organic carbon, and enhancing soil biodiversity to promote biological activity. Certain pests are better controlled under no tillage condition which favors a number of predators including ground beetles. Although reduced and no tillage practices are promising, they have not yet been fully adopted because of a number of constraints. One of the key constraints is weed control. In conventional agriculture, reduced tillage would necessitate increased utilization of chemical pesticides and fertilizers to maintain yields and control pests [91]. In organic farming, reduced tillage would mean increasing the machinery for weed control, therefore, increasing energy costs and labor time [92]. Redesigning of the cropping system with the introduction of such practices would be required to alleviate the constraints and increase deficiency. For example, it would be vital to rethink of the entire cropping system to better control weeds. This may entail modification of crop choice and crop rotations.

The mechanical soil disturbance encountered by AMF in tilled agricultural soils is not the same as that in natural ecosystems. For this reason, tillage has been found to be one of the key causes of alteration of AMF communities that associate with plant roots in agricultural systems [67]. Conventional tillage has a negative effect on mycorrhizal diversity, spore numbers, and mycorrhizal colonization [49, 93]. Consequently, there is likely to be a reduction in AMF effectiveness [94]. The extraradical mycelial network formed by AMF is destroyed as a result of periodically repeated mechanical soil disturbance. The network is a complex underground structure that represents a biological link for nutrient transport. Hence, it has been thought to be closely related to biomass production, biodiversity, and plant function [95, 96].

A comparison between frequently and infrequently tilled agroecosystems will clearly show this ecological shift in AMF communities [97, 98]. This observation may be accounted for by the fact that different AMF species exhibit different tolerance levels to hyphal disruption. Although spores can serve as a medium for AMF colonization, the process would require quite some time. A viable and well-structured underground mycelia network would result in faster root colonization since it facilitates AMF proliferation and hastens plant root penetration [99]. It has also been noted that AMF species differ with respect to their ability to restart colonization from fragmented root fragments or mycelium. Therefore, while intense tillage could favor AMF species that have the capacity to proliferate from fragmented roots, it could be detrimental to those species that are less able to proliferate in fragmented hyphae [100]. Glomeraceae species are a good example of AMF group that are abundant in tilled soil [101]. These species have the ability to form random hyphal connections after soil disruption. The Gigasporaceae family on the other hand does not regrow from hyphal fragments but rather utilizes spores as their main source of root colonization. Therefore, practicing minimal or no tillage by smallholder farmers would help in increasing AMF population and, hence, AMF symbiosis in SHS. Reduced or no till increases AMF colonization to both the current and succeeding crops. This is because the extraradical mycelium previously produced would act as a source of inoculum [93]. Until now, no study has been done to elucidate how different types of tillage practiced by smallholder farmers affect mycorrhizal networks and crop performance. It is thus necessary to conduct such field studies to identify the most appropriate tillage styles to be adopted in SHS to promote AMF symbiosis and overall crop productivity or performance.

## 9. Future Prospects

There is a need to examine crop cultivars among smallholders to determine their AMF compatibility and responsiveness. Consequently, the most responsive cultivars would be selected and supplied to smallholder farmers to help in forming AMF symbiosis. Most of the smallholder farmers have old landraces that could be tested to determine their AMF symbiosis efficiency. Breeding for mycorrhiza is another alternative that needs to be considered. AMF biodiversity patterns should also be mapped and their effectiveness determined in promoting various agroecosystem services. Moreover, there is need to promote on-farm conservation by use of “AMF nurseries” which entail small pieces of land ca 1 m^2^ cropped with highly mycorrhizal plants. Although beneficial fungi such as AMF play a fundamental role in producing important ecosystem services such as soil fertility, they have received little attention. Smallholder farmers are likely to adopt AMF and utilize them more widely if they are trained on the beneficial aspects associated with them. The starting point should be with model crops like forage legumes or cereals and later to other crops. To evaluate the potential role of AMF in improving soil fertility, research projects on exotic and native AMF species should be conducted and the best approach is participatory where smallholder farmers are actively involved.

Cheap inocula need to be availed through* in vitro* mass production, on-farm multiplication and use of nursery inoculated horticultural crops. Currently, AMF inocula are produced in various ways utilizing greenhouse, laboratory, or field-based methods. The greenhouse method entails inoculation of AMF spores into pots in which host plants are grown. The inocula obtained by these methods are commercially available, which implies that the costs of laboratory space/time and shipping must be borne by the farmer. On-farm production of inocula avoids most of the mentioned costs and could make AMF symbiosis and the associated environmental and economical benefits available to smallholder farmers. Also, these methods can produce inocula containing the indigenous AMF already adapted to one's farm.

## Figures and Tables

**Table 1:** Overview of recent studies showing different agroecosystem services provided by arbuscular mycorrhizal isolates to important crops among smallholder farmers.

AMF symbionts	Crop tested	Agroecosystem service	Conditions	References
*Funneliformis mosseae* and* Rhizophagus irregularis*	Chickpea (*Cicer arietinum *L.)	Increased plant biomass, yield and grain quality	Field	[13]

Mixture of* Glomus clarum, Gigaspora margarita*, and* Acaulospora* sp.	Coffee (*Coffea arabica *L.)	Protection of plants against phytotoxic effects of Cu and Zn	Greenhouse	[14]

Indigenous AMF consortium: *Glomus mosseae, G. fasciculatum, G. etunicatum, G. intraradices*, and *Scutellospora* sp.	Green pepper (*Capsicum annuum *L.), parsley (*Petroselinum crispum *(Mill.) Fuss), carrot (*Daucus carota *L.), and tomato (*Solanum lycopersicum* L.)	Increased plant and root biomass and yield quality	Greenhouse and field	[15]

Consortium: *G. intraradices, G. mosseae, G. aggregatum/intraradices, Acaulospora trappei, Entrophospora infrequens*, and *Glomus *sp.^*∗*^	Lettuce (*Lactuca sativa* L.) and Onion (*Allium cepa *L.)	Salinity tolerance	Greenhouse	[16]

*G. fasciculatum, G. clarum, G. etunicatum*, and *G. versiforme*	Long pepper (*Piper longum *L.)	Enhanced growth	Nursery and field	[17]

Consortium: *R. intraradices*, *G. aggregatum*, *G. viscosum*, *G. etunicatum*, and *G. claroideum*	Maize (*Zea mays* L.)	Improved crop growth, production, and grain quality	Field	[18]

Native AMF	Maize (*Z. mays*)	Increased uptake of K, Ca, and Mg	Field	[19]

*G. mosseae* and *G. intraradices*	Lucerne (*Medicago sativa *L.)	Increased glomalin-related soil protein (GRSP) and soil aggregate stability	Greenhouse	[20]

*G. mosseae, G. etunicatum, G. fasciculatum*, and *Gigaspora margarita*	Pepper (*C. annuum*)	Enhanced growth and control of *Phytophthora* blight	Pot, greenhouse, and field	[21]

*Glomus clarum*	Pepper (*C. annuum*)	Enhanced growth and fruit yield at high saline conditions	Glasshouse	[22]

On-farm produced *G. intraradices*	Potato (*Solanum tuberosum* L.)	Increased yields	Field	[23]

*G. etunicatum*	Soybean (*Glycine max* (L.) Merr.)	Improved growth in saline conditions	Greenhouse	[24]

*G. mosseae*	Soybean (*G. max*) and Lentil (*Lens culinaris* Medic)	Improved Zn uptake	Greenhouse	[25]

*G. intraradices, G. moss*e*ae*, and *G. etunicatum*	Strawberry (*Fragaria × ananassa *Duch.)	Improved productivity	Field	[26]

*G. intraradices*	Strawberry (*F. × ananassa*)	Improved fruit quality	Greenhouse	[27]

*G. moss*e*ae *and *G. hoi*	Sunflower (*Helianthus annuus *L.)	Increased yields	Greenhouse	[28]

*G. mosseae, G. intraradices*, and *G. coronatum*	Sunflower (*H. annuus*)	Weed suppression and increased P uptake	Greenhouse	[29]

Native AMF inoculum (consortium)	Tomato (*Solanum lycopersicum* L.)	Modifying plant response to Zn additions	Environment chamber	[30]

*G. intraradices*	Tomato (*S. lycopersicum*)	Suppression of root pathogens (*Alternaria solani*)	Climate chamber	[31]

*G. intraradices*	Tomato (*S. lycopersicum*)	Improved growth, production, and fruit quality under drought stress	Field	[32]

*G. mosseae*	Tomato (*S*. *lycopersicum*)	Suppression of root-knot nematode *Meloidogyne incognita*	Greenhouse	[33]

*G. intraradices*	Tomato (*S. lycopersicum*)	Improved salinity tolerance	Pot/greenhouse	[34]

*G. intraradices*	Tomato (*S. lycopersicum*) and Onion *(A. cepa*)	Improved yields	Field	[35]

*G. intraradices*	Tomato (*S*. *lycopersicum*)	Suppression of false root-knot nematode *Nacobbus aberrans*	Greenhouse	[36]

*G. intraradices*	Tomato (*S. lycopersicum*)	Enhancing growth, flowering, and yield	Field	[37]

*G. mosseae*	Tomato (*S. lycopersicum*)	Enhancing growth, flowering, fruit development, fruit yield, and quality	Growth chamber	[38]

*G. mosseae* and *G. intraradices* isolates and native AMF inoculum (consortium)	Clover (*Trifolium alexandrinum* L.) and maize (*Z. mays*)	Improved crop production and quality	Field	[39]

*G. etunicatum *and *G. mosseae*	Wheat (*Triticum aestivum* L.)	Improved growth, yield, and nutrient uptake	Field	[40]

*G. mosseae*, *G. hoi*, *G. etunicatum*, *A. scrobiculata*, and *A. *s*pinosa*	Yam (*Dioscorea rotundata*)	Increased tuber growth	Pot/greenhouse	[41]

Consortium of* G. mosseae*, *G. deserticola*, and *A. laevis*	Yam (*D. rotundata *and* D. alata*)	Increased nutrient uptake and yield	Greenhouse	[42]
